# Investigating Public Sentiment on Laryngeal Cancer in 2022 Using Machine Learning

**DOI:** 10.1007/s12070-023-03813-2

**Published:** 2023-04-26

**Authors:** Divya Rao, Rohit Singh, K. Prakashini, J. Vijayananda

**Affiliations:** 1 Department of Information and Communication Technology, Manipal Institute of Technology, Manipal Academy of Higher Education, Manipal, 576104 India; 2 Department of Otorhinolaryngology, Kasturba Medical College, Manipal Academy of Higher Education, Manipal, 576104 India; 3 Department of Radiodiagnosis and Imaging, Kasturba Medical College, Manipal Academy of Higher Education, Manipal, 576104 India; 4 Data Science and Artificial Intelligence, Philips, Bangalore, 560045 India

**Keywords:** Laryngeal cancer, Cancer awareness, Public sentiment, Artificial intelligence, Sentiment analysis

## Abstract

This study aims to investigate public sentiment on laryngeal cancer via tweets in 2022 using machine learning. We aimed to analyze the public sentiment about laryngeal cancer on Twitter last year. A novel dataset was created for the purpose of this study by scraping all tweets from 1st Jan 2022 that included the hashtags #throatcancer, #laryngealcancer, #supraglotticcancer, #glotticcancer, and #subglotticcancer in their text. After all tweets underwent a fourfold data cleaning process, they were analyzed using natural language processing and sentiment analysis techniques to classify tweets into positive, negative, or neutral categories and to identify common themes and topics related to laryngeal cancer. The study analyzed a corpus of 733 tweets related to laryngeal cancer. The sentiment analysis revealed that 53% of the tweets were neutral, 34% were positive, and 13% were negative. The most common themes identified in the tweets were treatment and therapy, risk factors, symptoms and diagnosis, prevention and awareness, and emotional impact. This study highlights the potential of social media platforms like Twitter as a valuable source of real-time, patient-generated data that can inform healthcare research and practice. Our findings suggest that while Twitter is a popular platform, the limited number of tweets related to laryngeal cancer indicates that a better strategy could be developed for online communication among netizens regarding the awareness of laryngeal cancer.

## Introduction

Laryngeal cancer, the cancer of the voice box, represents a third of all head and neck cancers [1]. Bad dietary habits, increased alcohol and tobacco consumption are risk factors of the cancer of the larynx [2]. Laryngeal cancer can cause a range of physical, emotional, and social challenges for patients and their families [1].

Twitter is a popular social media platform that allows users to post short messages called "tweets" of up to 280 characters [3]. Twitter usage varies from user to user and is used for sharing information, expressing opinions, etc. Information in Twitter, like any social media is shared in real-time. Hashtags are used to categorize tweets. A word that starts with a ‘#’ symbol is considered as a hashtag on twitter and is used for categorizing tweets. In recent years, social media platforms such as Twitter have become a popular means of sharing information, seeking support, and raising awareness about health issues, including cancer [4] Twitter provides a rich source of data that can be analyzed to gain insights into public sentiment, attitudes, and behaviors related to cancer.

In this study, we aim to investigate public sentiment on laryngeal cancer via tweets in 2022 using machine learning. Our research will leverage natural language processing [5] and sentiment analysis techniques [6] to classify tweets into positive, negative, or neutral categories, and to identify common themes and topics related to laryngeal cancer. By analyzing a large corpus of tweets, we hope to gain a better understanding of how the public perceives and discusses laryngeal cancer.

This research contributes to the development of targeted interventions and resources for individuals affected by laryngeal cancer. It helps to draw much needed attention to laryngeal cancer as a research area in general. Little work has been done with text mining of laryngeal cancer sentiments online. This study highlights the potential of social media platforms like Twitter as a valuable source of real-time, patient-generated data that can inform healthcare research and practice.

## Related Literature

The following section summarizes some recent research that uses AI sentiment analysis techniques on cancers.

Jun et al. [7] analyzed 6427 tweets about cancer and COVID-19 vaccines that were collected over a period of two years. They combined a variety of methods including text mining, manual coding and statistical analysis, and inductive qualitative thematic analysis. They were able to categorize groups of tweets by their users: cancer patients and caregivers and analyze if they were for or againt the covid 19 vaccination. With topic analysis, they analyzed vaccine hesitancy, self-reported adverse events, and COVID-19 disruption of cancer treatment as key themes.

Gereta et al. [8] explored “Global discussions about surgical cancer care on Twitter during COVID-19”. They were interested in the public perceptions about surgery as a treatment modality for cancers. Over 20000 tweets were collected that discussed various cancers such as breast, neurology, etc, over a period of two years. They predicted user demographic information using twitter account details. They analyzed information about user demographic, surgery costs, delay in care due to COVID 19 and other sentiments. They identified that surgery costs associated with cancer care was a common concern among patients who were active on Twitter.

Patel et al. [9] studied “Social Media Discussions About Colorectal Cancer During the COVID-19 Pandemic”. More than 72,000 tweets were extracted from January 2020 to April 2021 and were analyzed. They were extracted information about the gender, age, profession and businesses that tweeted about colorectal cancer. They also categorized topics of tweets such as death, cancer awareness, risk factors, detection and research tweets. They concluded that partnering with influencers may be an effective strategy for improving communication of future public health recommendations.

Naganathan et al. [10] performed a qualitative analysis of tweets related to breast cancer and COVID19. Their findings from the samplsize of 403 tweets showed that advocacy organizations tweeted the most about the topic and a majority of the tweets came from the USA. The categories and patterns that emerged from their work were patient hesitancy and vulnerability, increased efforts in knowledge sharing, and evolving best practices.

## Materials and Methods

### Data Requirement

For this research, a qualitative descriptive methodology was used to examine tweets collected from Twitter. These tweets were authored by a diverse range of individuals, including clinicians, health researchers, advocacy organizations, laryngeal cancer patients, and support persons. To comprehensively understand the experiences, perspectives, and challenges faced by people involved in laryngeal cancer care and those affected by laryngeal cancer, the researchers conducted a qualitative thematic content analysis of the tweets. This approach was selected to capture and analyze the expressions and viewpoints of these individuals.

### Inclusion and Exclusion Criteria

**Fig. 1:**
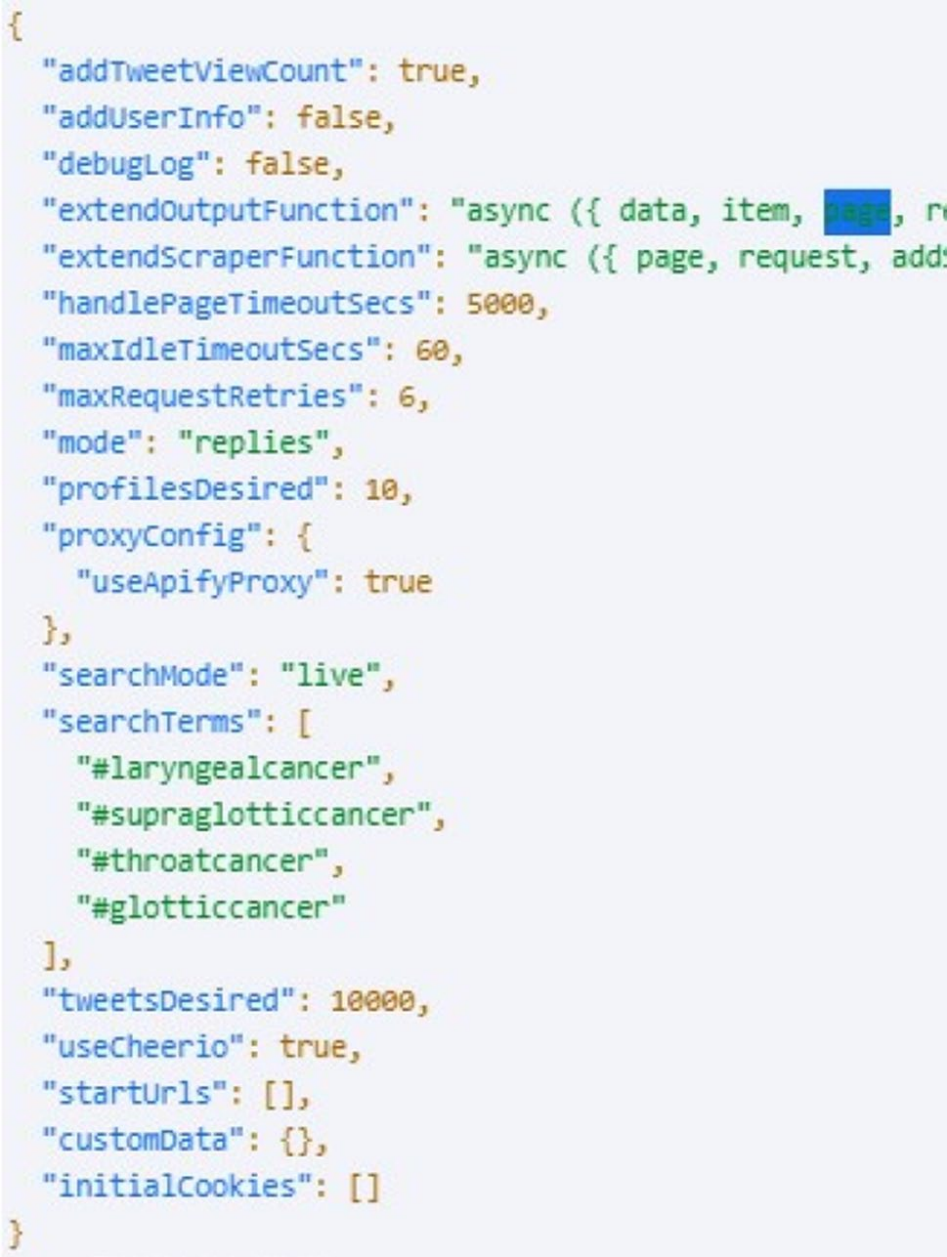
Parameters for data collection of laryngeal cancer tweets

The parameters set for extraction of Data were as follows: A The hashtags used for data retrieval were #throatcancer #laryngealcancer #supraglotticcancer #glotticcancer and #subglotticcancer with a date range search from 01 Jan 2022 to 01 Jan 2023. There was no filter on language or geolocation. All Tweet content was retrieved including images, videos and URLs. All keywords were retrieved. Retweet and like count for each tweet was not extracted. User profile information, such as their username, location, and bio were not extracted as this is an anonymous sentiment analysis study on Laryngeal Cancer. The scraped data was exported to a CSV format for further processing. The parameters of the Laryngeal Tweet retrieval are shown in Figure 1.

### Data Collection

All tweets were considered from 1st January 2022 to 20th February 2023 for this study. The total sample size for this study was 733 tweets. Data for this study was extracted from Twitter API via Apify [11] a tweet scraper. A tweet scraper is a tool or software that extracts data from Twitter, specifically tweets.

### Study Design

Retrospective

### Duration

1st Jan 2022 to 20th Feb 2023

### Sample Size

All tweets were considered in the specified time period. The total sample size for this study was 733 tweets.

### Data Preprocessing

The text cannot be directly analyzed for sentiment as it may contain information that is not useful for the final analysis. Therefore, it must undergo a series of preprocessing steps. The steps are described and an example tweet undergoing the series of transformations is subsequently described in Table 1.

- Step 1: Convert to Lower case

**Table 1:** Tweet Transformation after each preprocessing step

Original tweet text	#Coffee, the Beverage Is Associated to the Incidence of #LaryngealCancer, Scientists Suggest https://t.co/tr12owhy52
Step1: Convert to lower case	#Coffee, the beverage is associated to the incidence of #laryngealcancer, scientists suggest https://t.co/tr12owhy52
Step 2: Removal of hyperlinks	#Coffee, the beverage is associated to the incidence of #laryngealcancer, scientists suggest
Step 3: Removal of punctuation	Coffee the beverage is associated to the incidence of laryngealcancer scientists suggest
Step 4: Removal of stopwords	Coffee beverage associated incidence laryngealcancer scientists suggest
Final text	Coffee beverage associated incidence laryngealcancer scientists suggest

Sentiment analysis techniques can be case sensitive, therefore, to avoid misclassification, all the letters in the tweets are converted into lowercase. Any useful information is not lost.

- Step 2: Removal of hyperlinks

In this study, we do not use hyperlinks for our analysis. However, the tweets with links in them are present in our inclusion criteria. The link itself is not relevant to our analysis, however, the text before and after they hyperlink is important. Therefore, the second step is to remove the hyperlinks in the tweet, while keeping the remaining text intact.

- Step 3: Remove Punctuation

If a character is a punctuation mark, it is removed as punctuation marks do not add value to sentiment analysis. Question marks, brackets, hashtag symbols, exclamation points, periods, commas and semicolons are removed from the tweets.

- Step 4: Removal of StopWords

Stop Words are defined as words that carry little meaning on their own, such as "a", "an", "the", "and", "of", "to", "in", and "for". Removing stop words is beneficial in sentiment analysis tasks as it helps to reduce the dimensionality of the data and focus on the more meaningful words.

### Analyze Tweet Subjectivity

The preprocessed data was then scrutinized for subjectivity. Subjectivity quantifies the personal information in a text, in our case, the tweet. This value lies between 0 and 1. The higher the value, means that the tweet has more personal opinion than factual information. This score was computed for each tweet in the dataset.

### Analyze Tweet Polarity

The preprocessed data was also calculated for each tweet. For each word in a tweet, a score is assigned called the polarity score. This score is representative of the positivity/negativity of that particular word. The entire sentence then represents the summation of the individual polarities. This is also known as a sentiment score.

### Measure Word Frequencies

Measuring word frequencies is a common way to analyze twitter data. It helps us understand how often words are used in tweets on a particular topic and which words feature most commonly with the topic of interest, laryngeal cancer in our case.

## Results

The results of the sentiment analysis for 733 tweets related to laryngeal cancer are discussed below.

### Word Cloud Visualization

**Fig. 2:**
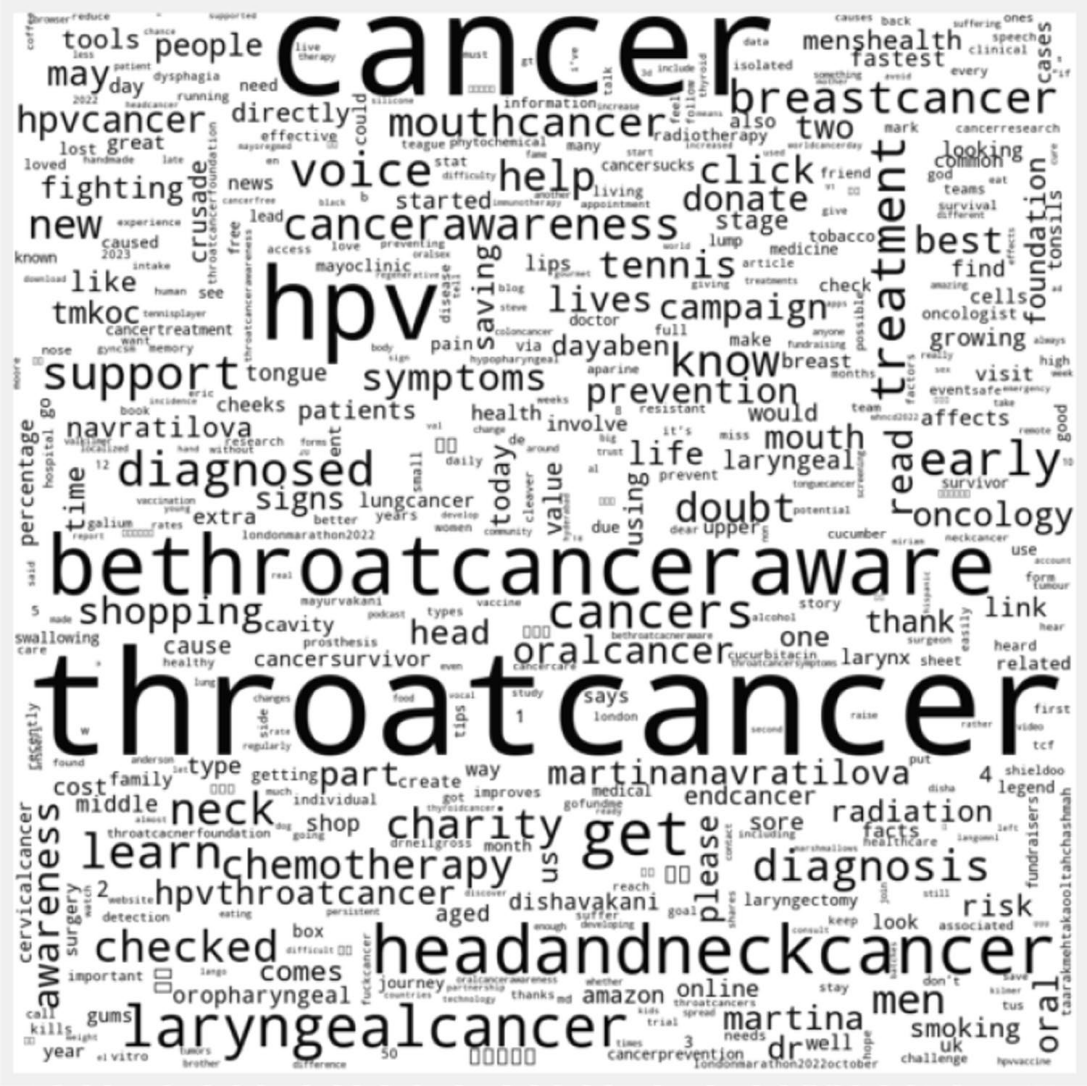
Wordcloud visualization of laryngeal cancer tweet dataset

Word cloud visualization is a visualization technique way to analyze sentiment distribution at a glance. Frequencies of all words in all the text corpus are computed. The words which have appeared in a large number of tweets have high frequency. When a word cloud is plotted, the size of words that appear is determined by the number of times the word has appeared in the corpus. Therefore, a frequently used word would appear bigger and bolder in the word cloud (Figure 2).

### Positive Tweets

After sentiment analysis was performed on the tweets, these are a few examples of tweets that were classified as positive by the sentiment analysis algorithm.

Sample 1 *Seann Wilkinson's friend was diagnosed with throat cancer several years ago. Thankfully they survived. He is now challenging himself to undertake the massive London to Brighton Cycle Race. Please give him some support. *https://t.co/Q8YAanhHMf* #ThroatCancer #BeThroatCancerAware *https://t.co/6zjhBiMlJ2

Sample 2 *An early detection of cervical cancer can save life. Consult us now. Call on—9881123006 Visit us—*https://t.co/r54rQhb07L* #ABMH #Ummeed #Oncology #cancer #lungcancer #throatcancer #cervicalcancer #cervix #brachytherapy #gynaeoncology #cancertreatment #treatment #healthylife *https://t.co/X49tKJlMd5

### Negative Tweets

Below are a few samples of tweets having negative sentiment as classified by the sentiment analysis algorithm.

Sample 1 *I'm very Saddened to Learn that @SimonCowell has #ThroatCancer #simonCowell has only two Year's To Live.. It's Desperately Sad. *

Sample 2 *We were amazed how he could look so much like #JimMorrison: Who is #ValKilmer? Unfortunately, Val Kilmer has been battling #throatcancer for some time. #Actors #MovieWorld *https://t.co/w4woI1wqlI

### Neutral Tweets

The following tweets were classified as having neutral sentiment by the model.

Sample 1 *Surgical resident and Masters student Dr Tim Lee presenting a systematic review and meta analysis on survival and toxicity with reduced dose radiotherapy in HPV associated OPSCC #throatcancer @anzhncs @Flinders @FlindersFound @AdelaideENTSurg *https://t.co/pOO1s6gNpI

Sample 2 *Rudra ENT Hospital OPD Time: 3 PM To 8 PM Details Information Please Visit Website: *https://t.co/10b5ttSZ00* Phone: 7000371689 #entdoctorbilaspur #entsurgeonbilaspur #ENTSpecialist #eardoctor #nosedoctor #throatcancer #throatdoctor *https://t.co/5YMHgy8fL3

### Tweet Analysis

**Fig. 3:**
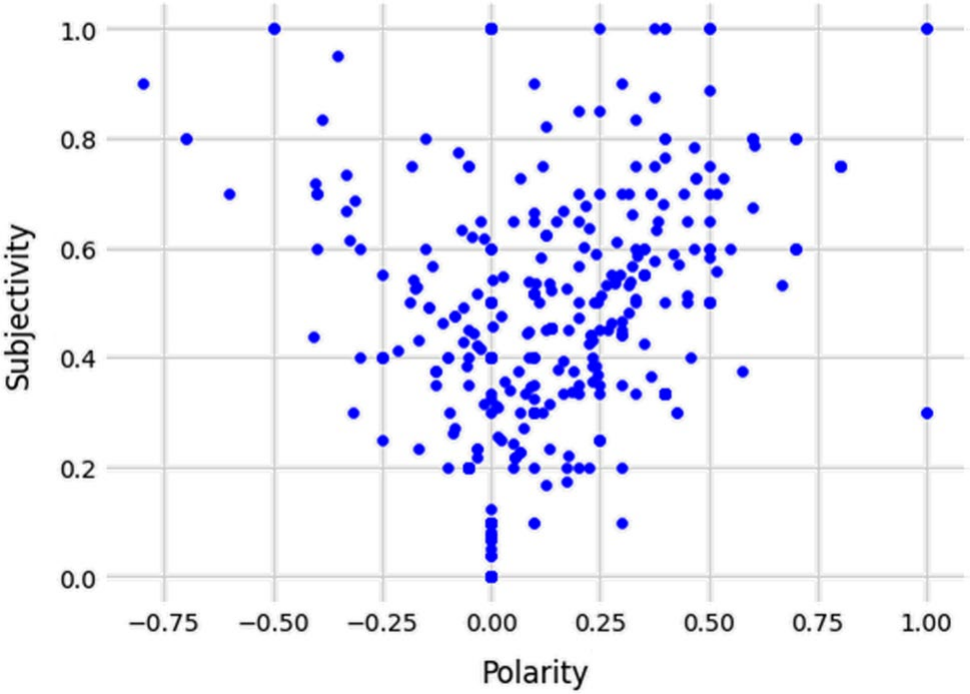
Sentiment analysis plot of each tweet in the dataset

**Fig. 4:**
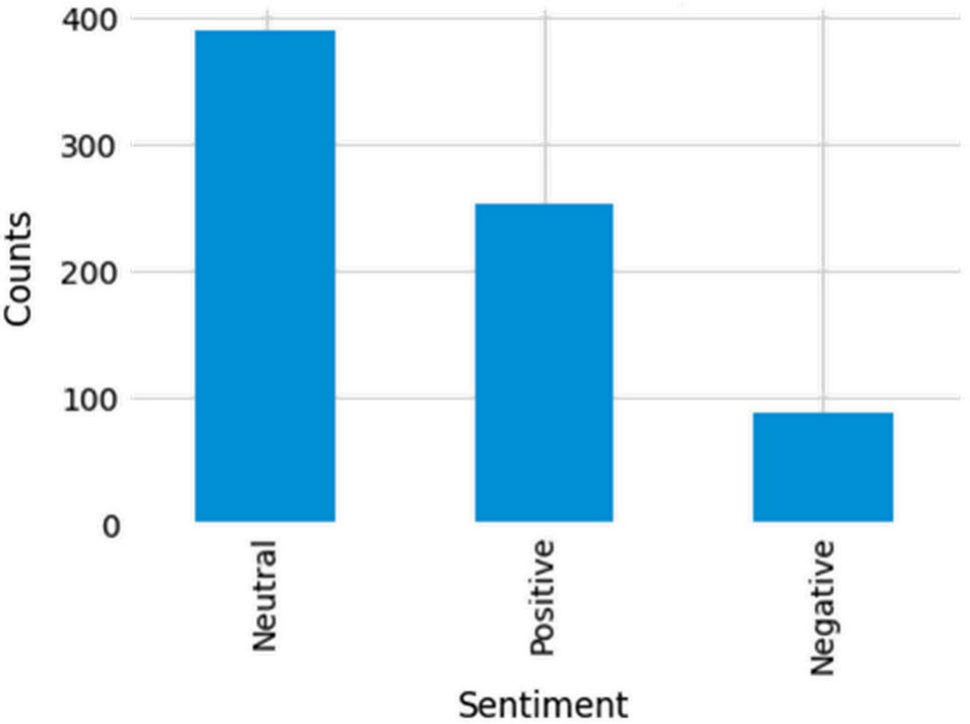
Counts of tweets with varied sentiments

The graph in Figure 3 shows us the distribution of sentiments in each tweet about laryngeal cancer in our corpus. A majority of the tweets have positive polarity. They represent positive sentiment of the user with respect to the topic. There are a lot of neutral tweets and relatively lower number of negative emotions detected in the tweet dataset. Subjectivity is represented along the y axis. The dataset has a good mix of opinions and facts and is not very factual or opinionated. 34.7% of the tweets have positive sentiment, 12.1% of tweets have negative sentiment and the rest of the tweets are neutral. Distribution of counts for each of the categories are illustrated in Figure 4.

## Conclusion

Our aim in this study was to analyze the public sentiment of laryngeal cancer on twitter over the past year. Since the dataset does not exist, we scraped all tweets related to laryngeal cancer and performed natural language processing on the dataset of 733 tweets. Laryngeal cancer was referred to as Throat cancer by Twitter users. Related studies that used twitter data to perform sentiment analysis found that COVID-19 was common theme that came up when other cancers such as colorectal cancer, breast cancer, cancer surgical care were mentioned. However, we did not find this to be true four our dataset. In our study, the topics that emerged were diagnosis, HPV, treatment, early detection, social media campaigns and tweets about famous people diagnosed with the disease. Site specific terminology like glottic, supraglottic and subglottic cancers were not mentioned. The overall sentiment of the tweets were neutral which came from authors publishing scientific articles about new research and from organizations advertising their products or services in cancer care. The opinioned, polarized tweets tended to come from public, especially patients and family or fans of celebrities who had contracted the disease.

This research can be extended by scraping a larger dataset from a larger timeline. User information and hashtag information can be collected and aggregated to estimate the overall purpose of the tweet such as support, education, cancer care, scientific discoveries, recovery, etc. This can provide further insight into social media discussions of laryngeal cancer on the internet. The study can be further expanded to include more social media sites other than twitter to get a larger pool of text to mine and analyze.

### Limitations

There are tweets with both English and non-English characters. The words containing non-English characters were removed and analyzed. Translation of the tweets in other languages would give us better insight into the sentiment of the tweet.

## Data Availability

The data used in this study was not used/published in any other publications.

## Code Availability

All the algorithms and libraries used for processing and analyzing the data have been cited in the paper.
